# The effects of art therapy on quality of life and psychosomatic symptoms in adults with cancer: a systematic review and meta-analysis

**DOI:** 10.1186/s12906-023-04258-4

**Published:** 2023-12-01

**Authors:** ShiShuang Zhou, MeiHong Yu, Zhan Zhou, LiWen Wang, WeiWei Liu, Qin Dai

**Affiliations:** 1 School of Nursing, Army Medical University, Chongqing, 400038 China; 2 Department of Gastroenterology, The Second Xiangya Hospital, Changsha, Hunan China; 3 Research Center of Digestive Disease, Central South University, Changsha, Hunan China; 4 Departments of Neonatology, People’s Hospital (ZhuHai Clinical Medical College of Jinan University), Zhuhai, China; 5 Department of Medical Psychology, Army Medical University, Chongqing, 400038 China

**Keywords:** Art therapy, Adults with cancer, Quality of life, Emotional symptom, Somatic symptom

## Abstract

**Background:**

Cancer-related psychological and physical disorders can mean stressful and painful experiences for patients. Art therapy, a form of complementary and alternative medicine, is an increasingly popular way to decrease emotional stress, alleviate somatic symptoms, and improve quality of life in patients with cancer. However, current systematic reviews have not explored the beneficial effects of art therapy. Moreover, there have been inconsistent findings on the effect of this therapy, and there is insufficient evidence to confirm the effects in adults with cancer. The objective of this study was to determine the efficacy of art therapy in improving quality of life and psychosomatic symptoms in adults with cancer.

**Methods:**

This systematic review and meta-analysis included adults with all kinds of cancer. Six English-language and three large Chinese-language databases were comprehensively searched for relevant studies. Gray literature and references were also checked. The quality of the included studies was evaluated using the Cochrane risk-of-bias assessment tool.

**Results:**

Eight eligible randomized controlled trials conducted in four countries were included. Art therapy improved overall quality of life, but had no significant effect on psychological health or physical health sub-dimensions in women with cancer. Moreover, art therapy alleviated anxiety and depression, but had only a tendency toward an effect on somatic symptoms.

**Conclusions:**

Moderate-quality evidence shows that art therapy is beneficial for women with cancer in terms of improving the overall quality of life and alleviating emotional symptoms (anxiety and depression). However, more high-quality randomized controlled trials are needed to determine the efficacy of this therapy on somatic symptoms.

## Background

Cancer is a very stressful and painful experience, and uncertain treatment results and the possibility of recurrence can cause anxiety, depression, and fear [1, 2]. Currently, the most common treatment strategy to increase survival rates involves a combination of therapies, including chemotherapy, radiation, and surgery. However, these methods have unwanted side effects, including fatigue, pain, and bad mood, which impact patients’ quality of life. The concept of quality of life is multidimensional and serves as a comprehensive indicator of the well-being of oncological patients, encompassing aspects of physical, material, social, and emotional well-being, as well as personal development and activity levels [3, 4]. Numerous assessment instruments are available for measuring the quality of life in cancer patients, and while there may be slight variations in the terminology, consistency is maintained between operational definitions and practical settings [5]. Recognizing that quality of life stands as a pivotal gauge of therapeutic effectiveness, complementary and alternative therapies, as supplementary approaches to standard medical treatments [4, 6], have progressively gained recognition for their role in enhancing the quality of life [7–9] and addressing psychosomatic symptoms, including two primary dimensions: somatic symptoms (fatigue, pain, loss of appetite) and emotional symptoms (anxiety and depression). [8, 10–12].

Art therapy is one main form of complementary and alternative therapy that patients with cancer are often offered in the clinic [13–15]. According to *About Art Therapy* (2018), published by the American Art Therapy Association, art therapy is a therapeutic modality applied over ongoing sessions with the assistance of a professional art therapist. The aim of art therapy is to improve cognitive and sensory-motor functions, cultivate emotional resilience, foster self-esteem and self-awareness, promote insight, enhance social skills, reduce and resolve conflicts and distress, and advance societal and ecological improvement [16].

With the mainstream literature, arts therapies are the therapeutic use of an art form, such as visual art, music, dance, drama and art therapy is the therapeutic use of the visual art form such as painting, paper cutting, and sculpture. During our literature retrieval process, we identified some systematic reviews have studied multiple kinds of arts therapies [17–19]. Some systematic reviews have confirmed the beneficial effect of sensory art therapies in people with cancer [20, 21]. Similar reviews have also focused on the effect of various art therapies on anxiety, depression, and quality of life [22, 23]. Therefore, it is difficult to identify the effect size of any specific art therapy and draw an exact conclusion from these publications. Several studies have indicated that art therapy is beneficial for people with cancer in various aspects, such as processing emotional stress [21, 24], alleviating emotional and somatic symptoms, and improving quality of life [25, 26]. For example, in people with breast cancer, art therapy has been found to alleviate negative emotions [27]. However, other studies have found no effect of art therapy on anxiety [28] or depression [28] in adults with cancer.

Given the findings described above, there is insufficient evidence about the specific effect of one kind of ars therapies on quality of life and emotional and somatic symptoms in adults with cancer. Thus, in this systematic review, we focused on one specific arts therapies—art therapy.

## Methods

We registered the protocol of this systematic review in PROSPERO (https://www.crd.york.ac.uk/prospero/) on November 11, 2019, with the ID number CRD42020156878. We followed the Preferred Reporting Items for Systematic Reviews and Meta-Analyses (PRISMA) guidelines for this systematic review.

### Information sources and search strategy

We searched for articles in electronic databases, including six English-language databases (PubMed, Medline, EMBASE, Cochrane, EBSCO PsycArticles, and Web of Science) and four of the largest databases in China (CNKI, Sinomed, Weipu, and Wanfang Data). To maximize the sensitivity of retrieval that is search as many related articles, when we searched, regardless of the interested outcomes, we just used the keywords of interested participants and the intervention method. The keywords were as follows: intervention containing therapies (art therapy; therapy, art; art therapies; therapies, art; art making; art production; painting (paint); drawing (draw); mandala)and participants (neoplasm; cancer; oncology; malignant; carcinoma; tumor; melanoma) but the search strategies were different for various databases. We settled the limitation or the key words in title or abstract only [tiab] included studies published in English or Chinese.

**Fig. 1:**
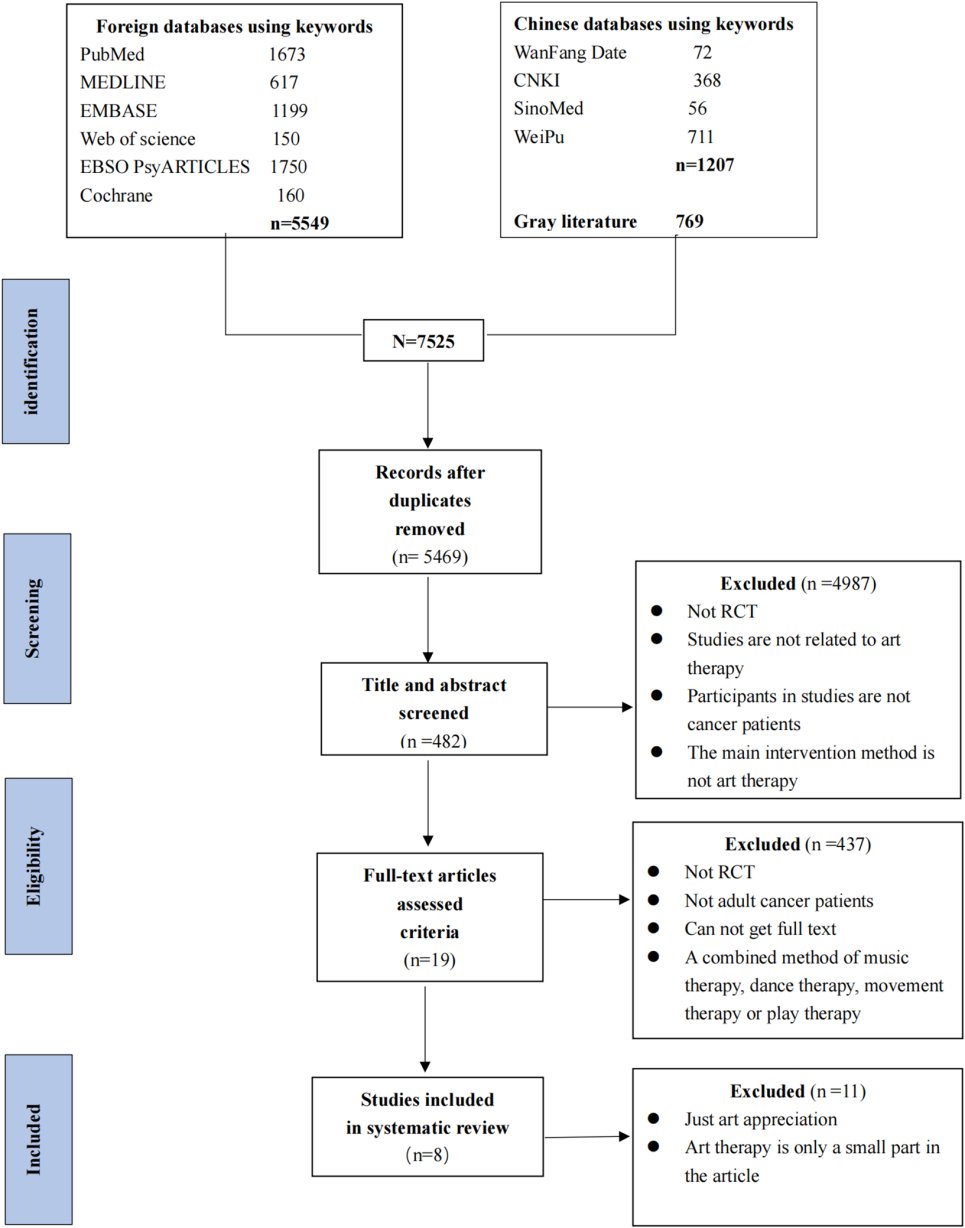
Flow diagram of the article retrieval process

For example, the search strategy for PubMed was as follows: (“art therapy” [MeSH Terms] OR “art therapy” OR “therapy; art” OR “art therapies” OR “art making” OR “art production” OR “mandala” OR “paintings” [MeSH Terms] OR “painting” OR “paintings” AND “paint” [MeSH Terms] OR “paint” OR “drawing” OR “drawings” OR “draw”) AND (“neoplasms” [MeSH Terms] OR “neoplasm” OR “neoplasia” OR “tumors” OR “tumor” OR “cancer” OR “cancers” AND “carcinoma” OR “malignant” OR “oncology”). Additionally, the references of our selected articles and some academic websites, such as Google Scholar, were checked to identify other potential articles. The process of searching was conducted on 22–23 March 2020 (updated on 20–22 March 2022). The publication date was restricted to the period from 1 to 1990 to the research date. Figure 1 shows the search strategy.

### Study selection

We imported all articles into the literature management software EndNote. After checking for and deleting duplicates, two researchers (ZSS and ZZ) selected studies independently based on the specified inclusion and exclusion criteria (see Sect. 2.2.1 and 2.2.2). First, the title was checked, followed by the abstract and the full text. The quality of each study was assessed after all steps had been completed. If there were any disagreements, a third researcher (YMH) made the final decision.

#### Inclusion criteria

The inclusion criteria were as follows: adults with cancer (aged ≥ 18 years). The types of interventions used included art therapy. The relevant indicators to demonstrate the effect were reported as follows: (1) quality of life, including overall quality of life and the two dimensions of psychological health and physical health, and/or (2) other symptom outcomes, including emotional symptoms (anxiety and depression) and somatic symptoms (fatigue and pain). Studies were randomized controlled trials (RCTs).

#### Exclusion criteria

The exclusion criteria were articles that reported intervention results of (1) arts therapies, (2) purely art appreciation, and (3) if the intervention included art therapy which was only a small part of the entire intervention.

### Methodological quality assessment

Because the studies included in this review were RCTs, we used Cochrane’s risk-of-bias assessment tool to evaluate the quality of the RCTs. All studies were critically appraised by two independent reviewers (ZSS and ZZ), and a third reviewer (YMH) resolved any discrepancies. All three reviewers agreed on the final outcomes of the assessment.

### Data extraction

Firstly, our research team constructed a standardized form containing the information we interested, including specific details about authors, countries, the year of publication, study design, participants, intervention details, outcome variables, data of the intervention effect with discussion. Then two different reviewers (WLW and LWW) read the full text of the included articles to extract information and fulfill the form. If there were disagreements, a third one (DQ) would participate into make decisions.

### Statistical analysis for meta-analysis

Review Manager 5 was used to organize the data. For risk of bias assessment, we imputed the results been assessed in the part of 2.3 (**Methodological quality assessment)** into the software and made the figure of risk-of-bias in the included studies.

Continuous variables were reported as the mean difference and standardized mean difference (SMD), binary variables as odds ratios, and relative risk or risk difference. The heterogeneity of multiple studies was tested using Cochrane’s Q before statistics were merged into effect size and I^2^, which represents the degree of heterogeneity. The appropriate models (fixed-effects or random-effects) were selected in line with heterogeneity; for example, when I^2^ < 50%, the fixed-effect model was applied to generate a pooled effect size. A non-significant Q-value of homogeneity means that the differences between studies were caused by sampling error, while a significant homogeneity result suggested that the differences between studies were not entirely a result of sampling errors, but also of other factors. According to Higgins’s interpretation of heterogeneity, an I^2^ of around 25%, 50%, and 75% indicates mean low, medium, and high heterogeneity, respectively.

Sensitivity analysis, or subgroup analysis, was used when necessary. Concerning the Cochrane Library’s recommendation, it is preferable to create funnel maps when including more than 10 articles. Thus, for this review, funnel maps were not appropriate, as only eight articles were included. Instead, the Cochrane Library’s Grades of Recommendation, Assessment, Development, and Evaluation (GRADE) criteria [29] were applied to assess the quality of the evidence of outcome variables. We evaluated the efficacy according to Cohen’s guidelines (whereby *d*-values of 0.2–0.4 indicate a small effect size, 0.5–0.7 indicates a medium effect size, and ≥ 0.8 a large effect size) [30].

## Results

### Selection results

The flow chart of the study selection process, shown in Fig. 1, was based on PRISMA. We found 6,756 articles in the databases and 769 in the gray literature. After an independent two-step selection by the two reviewers, eight articles were finally included. We extracted the main characteristics of these eligible studies and summarized them (Table  1) according to the Cochrane Handbook [29].

**Table 1:** Characteristics of included studies

	Reference	Country	Study Design	Paticipants	Intervention type,time	Intervention objective	Control	Measure Time	Main outcome	Evaluation tools	Grade
1	Thyme 2009	Sweden	RCT	Breast cancer patients uder radiotherapy Mean age = 59 years (Range: 37–69) IG: n = 20 CG: n = 21	Individual art therapy, 5 weeks	Express feelings and thoughts	Without intervntion	Week 0,8,16	Significant lower ratings of depression, anxiety, and somatic symptoms were reported for the art therapy group.	SCL-90: Axiety; Depression	High evidence
2	Monti 2006	USA	RCT	Adult female cancer patients Mean age = 53.6 years IG:n = 56 CG:n = 55	MBAT, 12 sessions 45 min each	Express their inner pain or feelings sufficiently	Wait-list controls	week 0,8,16	The MBAT group demonstrated a significant improvements in key aspects of quality of life after intervention	SCL-90-R: Axiety; Depression SF-36: Quality of life	Moderate evidence
3	Svensk 2008	Sweden	RCT	Women undergoing postoperative radiotherapy treatment for breast cancer IG: n = 20 CG: n = 21	Individual art therapy, 5 sessions 5 weeks, 1 h/week	Offer time and space for expression and reflection; give support; reduce stress and supporting agency	Without intervntion	week 0,8,24	A significant increase in total quality of life in the art therapy group.	WHOQOL-BREF; EORTC-QLQ-BR23: Quality of life	High evidence
4	Jang 2016	Korea	RCT	24 breast cancer patients Mean age = 51.58 ± 5.72 years IG: n = 12 CG: n = 12	MBAT, 12 sessions 45 min each	Express their inner pain or feelings sufficiently	Standard post-treatment clinic care	week 0,12	Depression and anxiety decreased, health-related quality of life improved significantly in the MBAT group	PAI: Depression, Anxiety EORTCQLQ-C30: Quality of life, fatigue	Moderate evidence
5	Monti 2013	USA	RCT	Women with brast cancer who were diagnosed beyond 6 months and winthin 3 years. IG::n = 98 CG::n = 93	MBAT, 8 weeks	Provide an nonverbal mode of identifying and organizing internal and external representations of stressors and related emotions	Breast Cancer Support Group control arm (BCSG)	week 1,9,16	MBAT is associated with significant, sustained benefits across a diverse range of breast cancer patients, particularly those with high stress levels	SCL-90: Anxiety, depression SF-36: Quality of life	Moderate evidence
6	Puig 2016	USA	RCT	Women with Stage I and Stage II breast cancer Mean age = 51.4 ± 11.9 years IG: n = 20 CG: n = 19	Individual creative art therapy, 4 weeks, 60 min each	To provide an opportunity for emotional expression and support	A delayed treatment of intervention	week 0,4	Participants’ negative emotional states were improved in intervention group	POMS: Dpression, fatigue	Moderate evidence
7	ShuFen,Z 2017	China	RCT	Cancer patients (male: n = 41; female: n = 45) IG: n = 43 CG: n = 43	Group art therapy, 6 weeks	To express their emotions	Basic nursing care and pain care	week 0,2,4,6	Mandala can decrease cancer patients’ anxiety	Self-rating anxiety scale (SAS): Anxiety	Moderate evidence
8	YuQiao,S 2017	China	RCT	Breast cancer patients who need to accpet operation (Mean age: 38.2 ± 3.6 years) IG: n = 115 CG: n = 115	Individual painting art therapy, After hospitalized 1–2 days, before and after operation	Establish well doctor-patient relationship and help patients release pressure through painting art therapy	Usual care after breast cancer surgery	week 0 After 1 months and 3 months after operation	Painting art therapy can improve patients’ quality of life	Cancer patients quality of life scale (self-make): Quality of life	Low evidence

SCL-90: the Symptom Check List–90; MBRT: Mindfulness-based art therapy; SCL-90-R:the Symptoms Checklist Revised(Derogatis, 1993); SF-36: Medical Outcomes Study Short-Form Health Survey; The WHOQOL-BREF: the Swedish version of the WHO instrument WHOQOL-BREF; The EORTC-QLQ-BR23: of the European Organization for Research and Treatment of Cancer instrument, EORTC Quality of Life Questionnaire, version 1.0.; PAI: Personality assessment inventory; the EORTC-QLQC30: European Organization for Research and Treatment of Cancer Quality of Life Questionnaire; POMS: the Profile of Mood States

### Types of participants

In total, 721 participants from four countries, namely China (n = 316), the USA (n = 341), Sweden (n = 42), and Korea (n = 24), were included in this review. However, the 42 Swedish participants came from the same study, which was reported in two separate articles [31, 32]. While our strategy was not to select participants with one specific type of cancer, participants from seven of the eight studies were all people with breast cancer (n = 593), and another sample consisted of patients with a variety of types of cancer. Furthermore, only one study from China included men [33] (n = 41). Participants in all the other studies were female. Notably, participants were not co-diagnosed with other disease such as cancer with a mental health co-diagnosis.

### Types of intervention

Table 1 shows the details of the interventions. The intervention methods of the included studies were primarily based on art therapy in group or individual sessions. The therapies lasted from 4 to 12 weeks, and almost all intervention instructors were qualified art therapists. However, in China, a system for qualification authentication for art therapists has not been established. In the Chinese studies, counseling psychologists or psychotherapists were recruited to conduct art therapy. The control group of some studies comprised a wait-list art therapy group who were receiving a regular care intervention. However, only one art therapy course in China consisted of two sessions per week [33], while the others included interventions once a week. In addition, Monti et al. [28, 34] used a type of art therapy called mindfulness-based art therapy, which combined art therapy with mindfulness therapy in intervention studies conducted in 2006 and 2013. The control group received a delayed intervention in 2006 and mindfulness-based stress reduction therapy in 2013.

### Outcome measures

The main indicator was quality of life and its sub-dimensions (psychological health and physical health). These variables were assessed using the European Organization for Research and Treatment of Cancer Quality of Life Questionnaires, the Short-Form Health Survey, and the World Health Organization Quality of Life Questionnaires. Perceived emotional and somatic symptoms were primarily measured by the Symptoms Check List-90 (anxiety, depression, and fatigue), the Profile of Mood States (depression and fatigue), and the Personality Assessment Inventory (depression and anxiety). Table  1 contains detailed information about the included studies.

### Risk-of-bias in the included studies

**Fig. 2:**
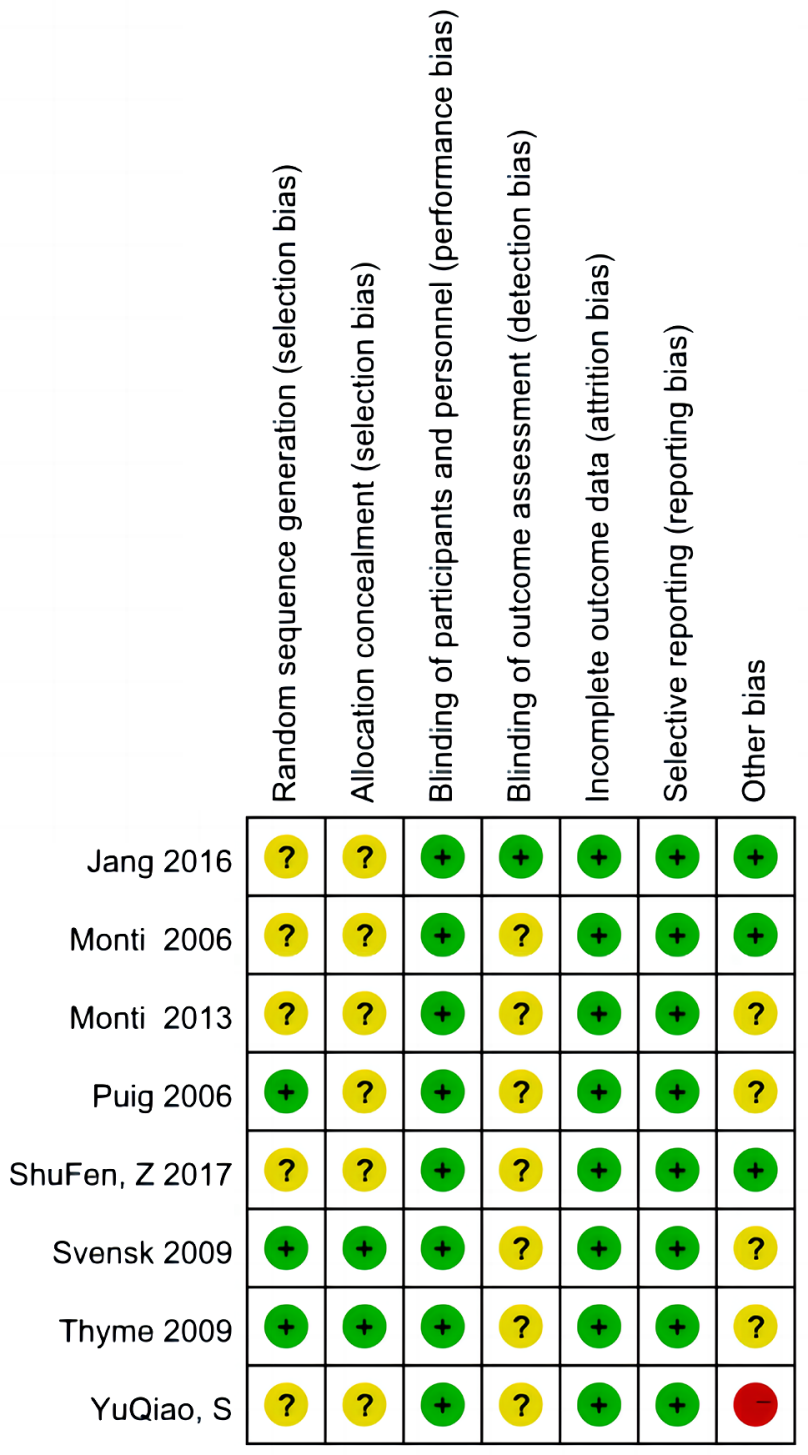
The risk-of-bias assessment for each of the included studies

Figure 2 shows the risk-of-bias assessment for the eight included studies. All studies were RCTs, which should have been conducted with enough rigor to be able to apply the randomized assignment sequence method. However, some studies did not report the randomization generation method [28, 34, 35]. Although a random allocation square can conceal imperfections in the study and could break predetermined and predictable allocation sequences [36], only two of the eight studies took note of the concealment of a random allocation square [31, 32]. Blindness makes art therapy impossible for participants. Therefore, all included studies were evaluated to indicate a low risk of blindness among participants. While it was feasible to perform an outcome assessment of blindness, almost no studies mentioned blindness in the outcome assessment or data processing.

It is understandable that in intervention studies, a certain number of participants drop out. However, YuQiao et al. [37] recruited 230 participants in their study, and no participants dropped out. Such a perfect follow-up of participants is questionable. The Cochrane Handbook [29] states that if there are missing data, the authors must explain in detail the reason for the discrepancy and how they dealt with it when preparing a paper for publication. The majority of the studies included in our review did not perform well concerning attrition bias.

No studies tended to report data selectively. With regard to other biases, the detailed baseline information of most studies was not reported. However, the demographic data of the participants were compared. The outcome indicators of two studies [31, 38] were not compared at baseline. For example, Thyme et al. [31] stated that the outcomes of the two groups were approximately the same at baseline, but the P-value was not reported in the main text.

### Quality grade of the included studies

With respect to the quality of the included RCTs in GRADE, only two studies [31, 32] performed well in random sequence generation and allocation concealment and were considered high-quality evidence. YuQiao et al.’s [37] evidence was graded as low quality due to the high risk of other bias, unclear selection, and detection bias. Monti et al.’s [28] study, which provided moderate-quality evidence, was not included in the meta-analysis because the control group (standardized mindfulness-based stress reduction) was different from those in the other studies (wait-list or usual care), and the data were incomplete. The RCT by Jang et al. [35] provided moderate-quality evidence given its unclear selection bias, even though they noted that their outcome measurement assessment was blinded. The remaining three papers were also considered to present moderate-quality evidence. Overall, the grade quality of the studies included in our review was rated as moderate.

### Effect of art therapy

#### Quality of life

##### Overall quality of life

**Fig. 3:**
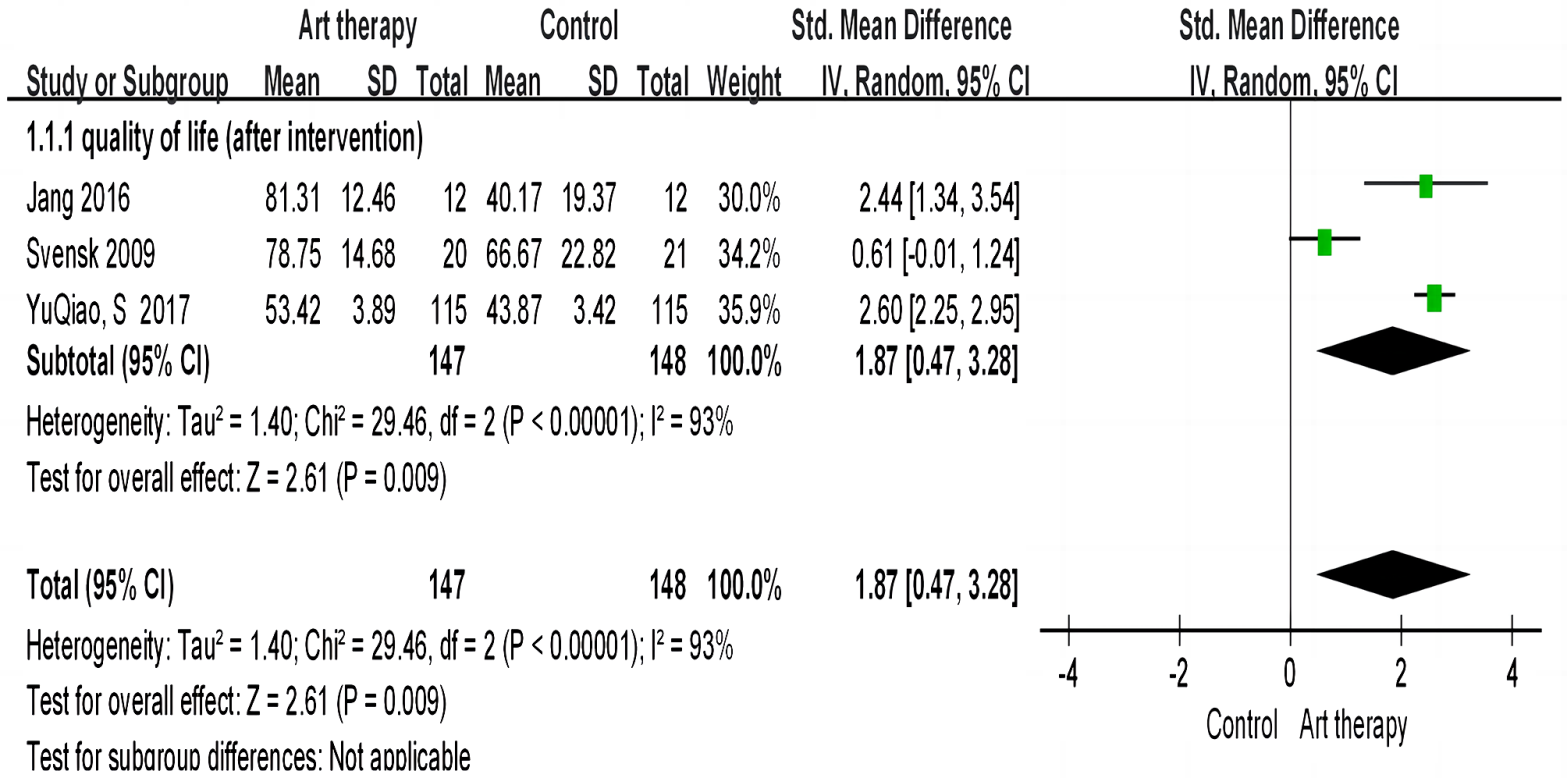
Effect of art therapy on overall quality of life in people with cancer

Figure 3 shows a random-effect meta-analysis of the impacts on the overall quality of life after art therapy intervention. We found a significant effect of art therapy on quality of life, with a large effect size (SMD = 1.87; 95% CI = 0.47 to 3.28; p = 0.009). A high heterogeneity between studies was also found (x2 = 38.89; p < 0.00; I2 = 92%).

##### Sub-dimension of quality of life: psychological health

**Fig. 4:**
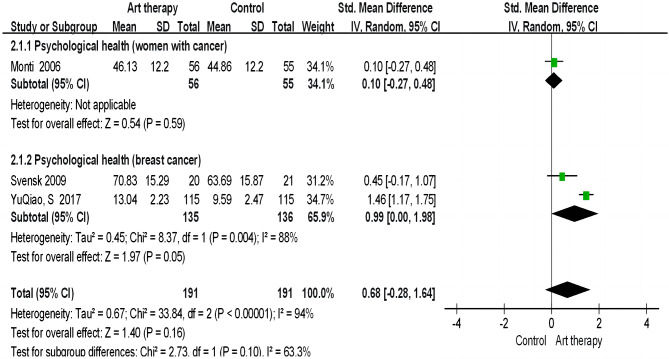
Effect of art therapy on psychological health in people with cancer

Referring to the “psychological health” sub-dimension of quality of life, the results revealed no significant difference between the intervention and control groups (pooled effect SMD = 0.68; 95% CI = − 0.28 to 1.64; *p* = 0.16; Fig. 4). The heterogeneity of three studies (x^2^ = 2.73, *p <* 0.00, I^2^ = 94%) may have come from sampling errors or the use of different measurement instruments.

##### Sub-dimension of quality of life: physical health

**Fig. 5:**
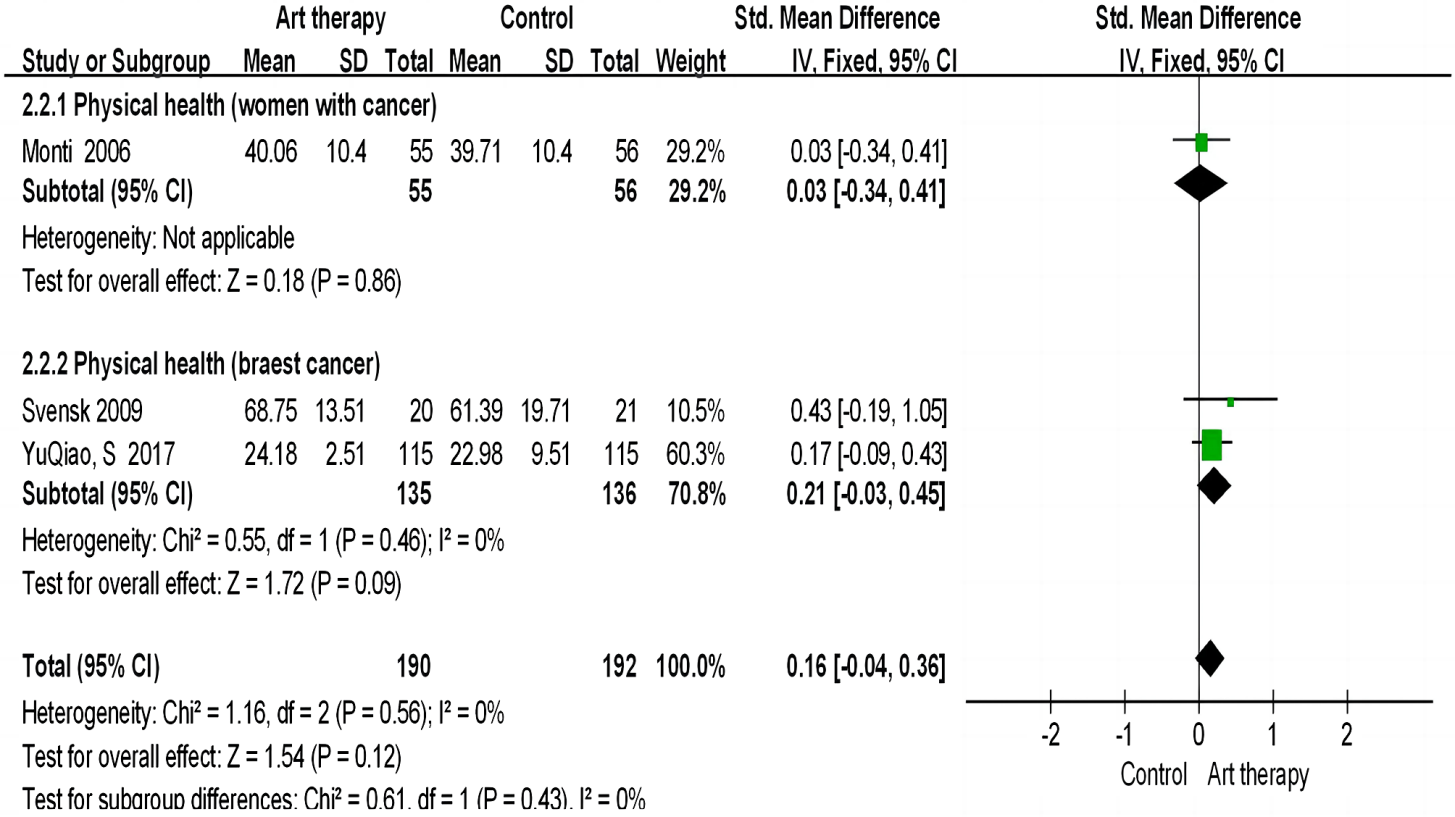
Effect of art therapy on physical health in people with cancer

Referring to physical health, which is another sub-dimension of quality of life, we found that art therapy had no significant effect on the physical health of women with cancer (SMD = 0.16; 95% CI = − 0.04 to 0.36; *p* = 0.12; Fig. 5); heterogeneity between studies was found (x^2^ = 1.16; *p* < 0.00; I^2^ = 0%; Fig. 5).

#### Psychosomatic symptoms

##### Anxiety

**Fig. 6:**
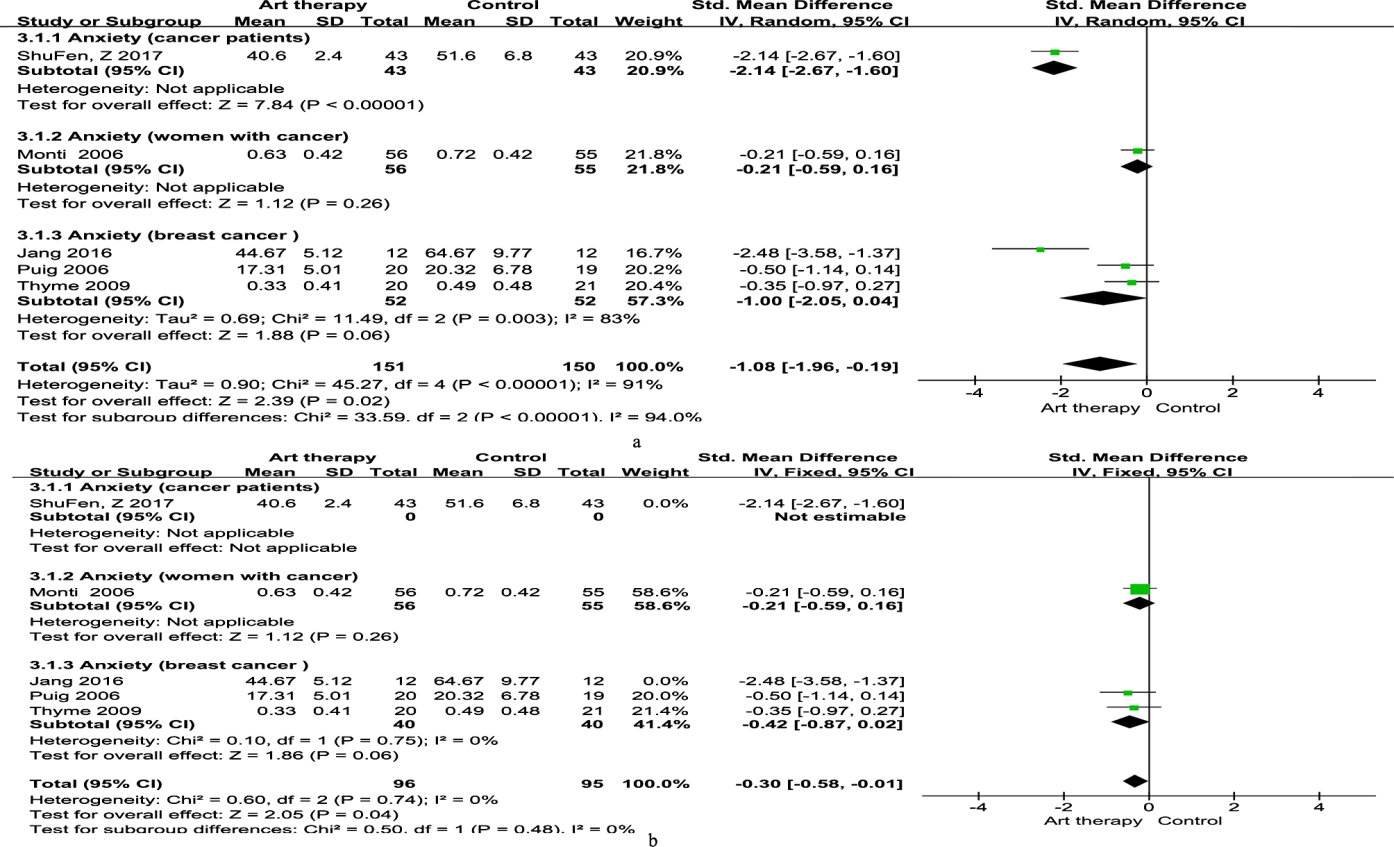
Effect of art therapy on anxiety in people with cancer. (**a**) Subgroup analysis; (**b**) Sensitivity analysis

The overall effect size of intervention on anxiety was − 1.08 (95% CI = − 1.96 to − 0.19; *p* < 0.00; Fig. 6). This significant result indicated that art therapy could moderately reduce anxiety in patients with cancer, especially those with breast cancer. There was heterogeneity among studies (x^2^ = 45.27; *p <* 0.1; I^2^ = 91%). The sensitivity analysis indicated that after removing the studies by Jang et al. [35] and ShuFen et al. [33], the heterogeneity among studies reduced dramatically to 0%, but the significance of art therapy overall was unaffected (SMD = − 0.30; 95% CI = − 0.58 to − 0.01; *p* = 0.04), resulting in moderate-quality evidence and small effect size.

##### Depression

**Fig. 7:**
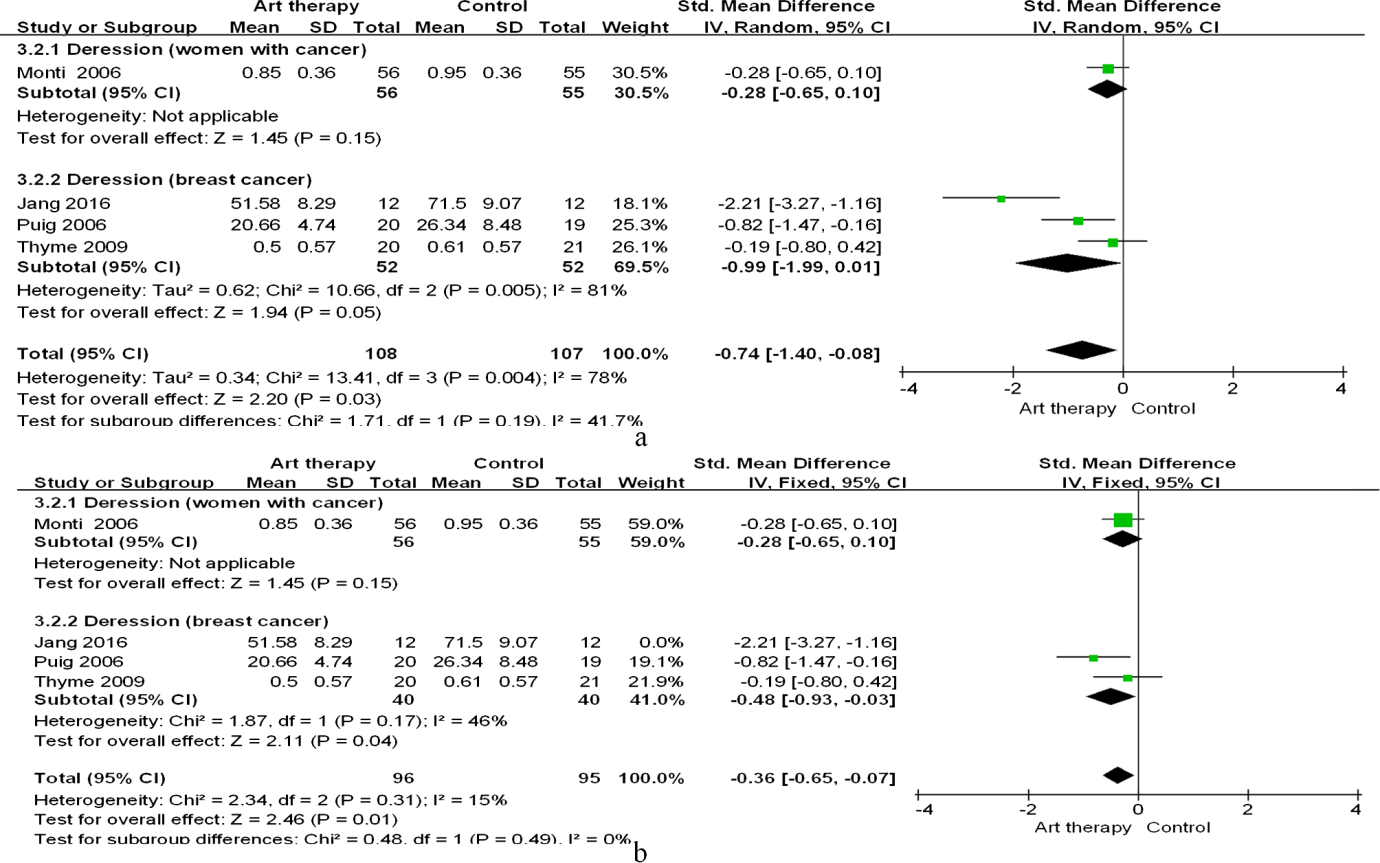
Effect of art therapy on depression in people with cancer. (**a**) Subgroup analysis; (**b**) Sensitivity analysis

Four studies generated a pooled effect size (SMD = − 0.75; 95% CI = − 1.40 to − 0.11; *p* = 0.02; Fig. 7), whereby art therapy was found to reduce depression in women with cancer. Heterogeneity was observed among studies (x^2^ = 13.41; *p <* 0.00; I^2^ = 78%). The sensitivity analysis indicated that after the removal of Jang et al.’s (2016) study, the heterogeneity among studies reduced from 78 to 15%. This also affected the significance of art therapy overall (SMD = − 0.39; 95% CI = − 0.68 to − 0.11; *p* = 0.007), which resulted in moderate-quality evidence and small effect size.

##### Somatic symptoms

**Fig. 8:**
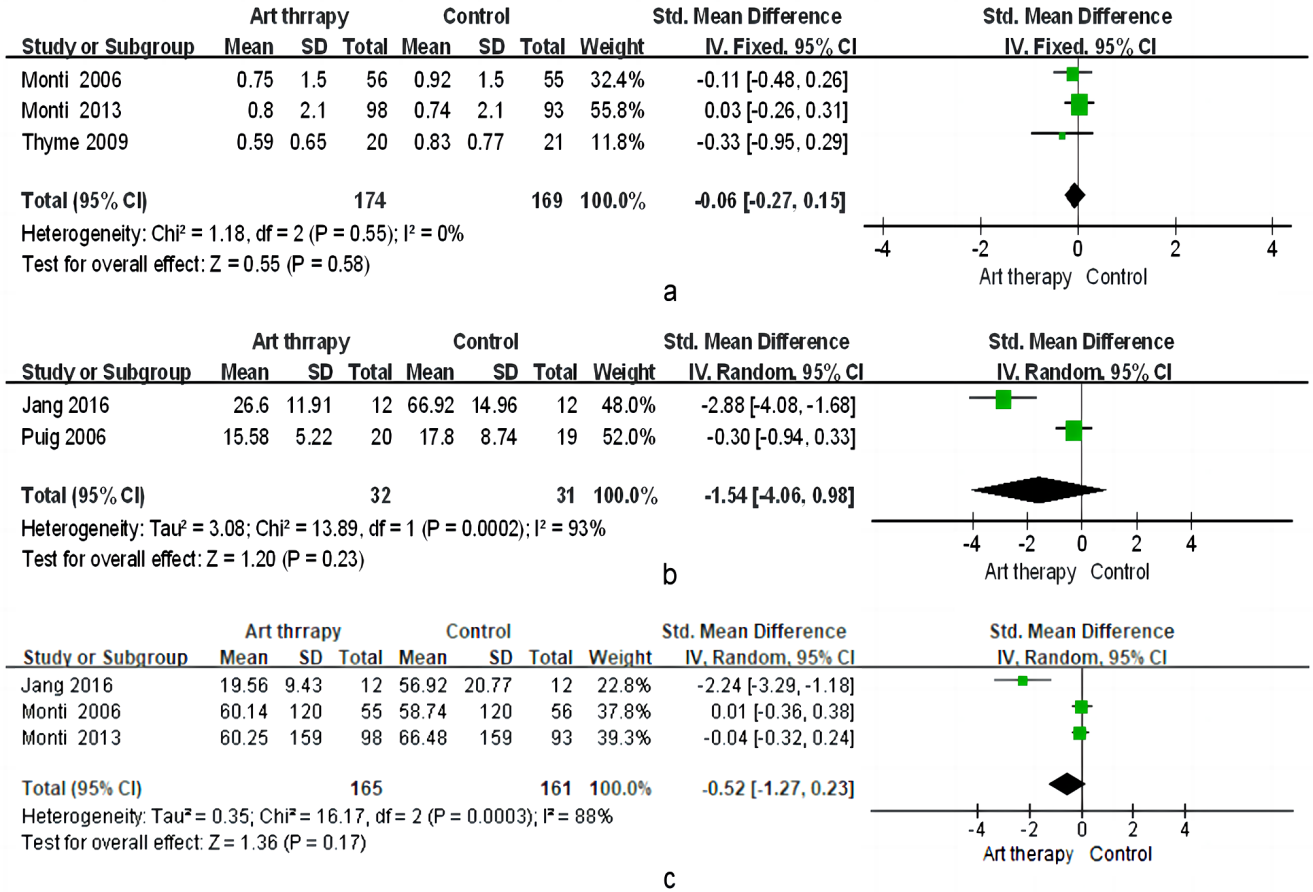
Effect of art therapy on somatic symptoms, fatigue, and pain in people with cancer. (**a**) Somatic symptoms; (**b**) Fatigue; (**c**) Pain

As Fig. 8a shows, art therapy had no significant effect on somatic symptoms (SMD = − 0.06; 95% CI = − 0.27 to 0.15; *p* = 0.58), and no heterogeneity was found between the three studies (x^2^ = 1.18; *p* = 0.55; I^2^ = 0%). Two studies that included women with cancer and fatigue were included in our analysis, which suggests that there was no significant effect of the intervention on fatigue (SMD = − 1.54; 95% CI = − 4.06 to 0.98; *p* = 0.23; Fig. 8b). Heterogeneity among studies was found (x^2^ = 13.89; *p <* 0.00; I^2^ = 93%). Three studies found that the intervention reduced pain (SMD = − 0.52 (Fig. 8c and 95% CI = − 4.06 to 0.98, *p* = 0.17), which did not achieve a significant effect with heterogeneity (x^2^ = 16.17; *p <* 0.00; I^2^ = 88%).

## Discussions

This meta-analysis confirmed that art therapy was beneficial for women with cancer. Moderate-quality evidence revealed that this therapy improved overall quality of life and alleviated emotional symptoms (anxiety and depression).

### Effect of art therapy

#### Quality of life

In total, three articles were included to assess the effect of art therapy on the quality of life of women with cancer and indicated that this intervention promoted overall quality of life, with a large effect size (SMD = 1.83). art therapy did not improve the sub-dimensions of quality of life (psychological health and physical health). This may be because the effect of the therapy on sub-dimensions was small, or there were not enough included studies for each sub-dimension. Therefore, the effect on separate dimensions did not reach significance, but the integrated effect on the overall quality of life did.

The above results suggest that painting-based therapy can be applied as a form of complementary and alternative therapy in clinical cancer treatments to improve overall quality of life, a key indicator for the curative effect of cancer treatment [7, 39]. Thus, during their clinical cancer treatment, women might have better outcomes by receiving a combination of traditional therapy and art therapy. However, another systematic review found that the effect of art therapies on overall quality of life did not reach significance (SMD = 0.15; 95% CI = − 0.09 to 0.40; *p* = 0.22) [40]. These conflicting results may stem from differences in the various art-intervention methods and/or the target population (e.g., male or female patients). From this, it can be concluded that more evidence is still needed to confirm the effect of various art therapies on quality of life. However, the results demonstrate that painting-based intervention has an effect on overall quality of life in women with cancer. This finding could be strengthened by qualitative evidence [32], especially given that the World Health Organization has defined quality of life as an individual’s own perception of their position in life.

#### Emotional symptoms

Our meta-analysis confirmed that art therapy reduced anxiety and depression in women with cancer. All the included articles indicated large (SMD = − 1.08) and moderate (SMD = − 0.74) effect sizes regarding the effects on anxiety and depression, respectively, which correspond with the results of another review on the effects of art therapy [18]. Moreover, when we excluded two large-sample studies [33, 35] from the anxiety analysis and one large-sample study [35] from the depression analysis, the effect sizes were small. Additionally, the heterogeneity dropped from 91 to 0% for anxiety and 78–15% for depression, which may have resulted from the different effect sizes between the included studies.

#### Somatic symptoms

In this systematic review, the pooled effect of art therapy on somatic symptoms was not significant. However, in the single experimental studies [28, 34, 38], art therapy did have a positive effect on these indicators. In addition, we found no benefits of art therapy on fatigue or pain in women with cancer, which suggests that art therapy may have little effect in the management of fatigue and pain. However, some quasi-experimental studies reported that art therapy helped patients with cancer deal with fatigue [41, 42]. Future investigations are needed to determine the effect of art therapy on somatic symptoms.

Quality of life can be affected by many factors, such as psychological health, physical health, emotional symptoms, somatic symptoms, social functions, and environment [40]. The positive effect of art therapy on overall quality of life might have been mainly driven by the improvement of anxiety and depression, especially as the core aim of art therapy is to offer a way for patients to express their inner feelings sufficiently [40]. This result is consistent with the idea that art therapy is a type of mind-body intervention that supports the power of the mind, which in turn influences the body [17]. Thus, with art therapy, women with cancer can cope with negative emotions, experience psychological growth, and achieve a better quality of life.

### Further implications

Notably, most studies included in this systematic review were conducted on women with breast cancer. This corresponds to other reviews, which have observed that women with cancer receive more psychological support than males, especially those with breast cancer [18, 19, 40]. However, men who are diagnosed with cancer also experience psychological stress. Moreover, men with cancer are more likely to be stoic, to have a psychological status that is resistant to change, and to be inarticulate about their emotions [43]. Wood et al. [40] suggested that the uptake of art therapy by men should be more acceptable than other types of psycho-social support. The gender gap in the application of art therapy needs to be recognized by researchers and clinical staff. Given that the findings of this systematic review were based on data from, predominantly, participants with breast cancer or female patients with cancer (rather than all types of cancer), an appropriate conclusion is that the effect of art therapy was confirmed in women with cancer, but more evidence is needed to make the same conclusion in men with cancer.

According to the included studies, the advantages of art therapy are not limited by place, time, race, or age. People with cancer aged up to 87 years [28] and of different races [44] seem to benefit from art therapy. Moreover, studies from four countries were included in our meta-analysis, which shows that the results are generalizable and not restricted to just one country. Furthermore, art therapy is more convenient, economical, and practical than traditional forms of therapies, as it can be practiced with simple materials such as paper and paints. However, it may require the input of qualified instructors to achieve optimal effects.

In conclusion, qualified art therapists, who have undergone systematic training, are capable of devising appropriate intervention plans for target populations. For instance, in the United States, art therapists typically complete an association-certified Master’s program in a counseling-related specialty, followed by mandatory internships and supervised therapy sessions (arttherapy.org/). This underscores the importance of engaging qualified professionals for optimal benefits in art therapy interventions. However, it is crucial to note that this should not serve as a barrier to applying art therapy in cases where qualified therapists are not readily available. Art itself possesses inherent therapeutic potential. From the types of intervention in results, we found that although the processes and details of the art therapy differed between the included studies in this review, the aim of the intervention was similar in all of them—to help participants express themselves, implicating that key factor for the effectiveness of art therapy is expression. Future research could explore the development of standardized art therapy interventions, facilitating their procedural and accessible application by healthcare providers and thus promoting wider adoption.

### Limitations

This study has some limitations that should be noted. First, a limited number of RCTs was included, so certain subgroup analyses could not be performed. Second, limited studies with outcome variables and subgroups may not be representative of the actual overall effect. Moreover, although all included studies were RCTs, few implemented allocation concealment and blinded outcome assessments, thereby decreasing the quality of the final results.

## Conclusions

This meta-analysis confirmed that art therapy is beneficial for women with cancer, with moderate-quality evidence of benefits for overall quality of life, anxiety, and depression. However, we found insufficient evidence for the ability of art therapy to alleviate psychological health, physical health, and somatic symptoms. The results of this study must be interpreted with caution given the heterogeneity in the forms of art therapy, time measurements, instruments, regions, and risk-of-bias in the included studies. Thus, researches into the effects of art therapy are still in infancy. More high-quality RCTs that focus on people with different types of cancer, other than breast cancer, and that include both male and female patients are needed to strengthen the evidence. Furthermore, future work should consider the effect of one specific type of art therapy and explore its effects on meaningful clinical outcomes.

## Data Availability

The datasets generated and/or analyzed during this study are available at PubMed, Medline, EMBASE, Cochrane, EBSCO PsycArticles, and Web of Science; Chinese databases (CNKI, Sinomed, Weipu, and Wanfang Data).
